# Cytokeratin 19 *(KRT19)* has a Role in the Reprogramming of Cancer Stem Cell-Like Cells to Less Aggressive and More Drug-Sensitive Cells

**DOI:** 10.3390/ijms19051423

**Published:** 2018-05-09

**Authors:** Subbroto Kumar Saha, Kyeongseok Kim, Gwang-Mo Yang, Hye Yeon Choi, Ssang-Goo Cho

**Affiliations:** Department of Stem Cell and Regenerative Biotechnology, Incurable Disease Animal Model & Stem Cell Institute (IDASI), Konkuk University, Seoul 05029, Korea; subbroto@konkuk.ac.kr (S.K.S.); proproggs@naver.com (K.K.); slayersgod@nate.com (G.-M.Y.); hyeon.choi24@gmail.com (H.Y.C.)

**Keywords:** *KRT19*, cancer stem cell, sphere formation, cell migration, drug-resistant, cancer stem cell reprogramming

## Abstract

Cytokeratin 19 (*KRT19*) is a cytoplasmic intermediate filament protein, which is responsible for structural rigidity and multipurpose scaffolds. In several cancers, *KRT19* is overexpressed and may play a crucial role in tumorigenic transformation. In our previous study, we revealed the role of *KRT19* as signaling component which mediated Wnt/NOTCH crosstalk through NUMB transcription in breast cancer. Here, we investigated the function of *KRT19* in cancer reprogramming and drug resistance in breast cancer cells. We found that expression of *KRT19* was attenuated in several patients-derived breast cancer tissues and patients with a low expression of *KRT19* were significantly correlated with poor prognosis in breast cancer patients. Consistently, highly aggressive and drug-resistant breast cancer patient-derived cancer stem cell-like cells (konkuk university-cancer stem cell-like cell (KU-CSLCs)) displayed higher expression of cancer stem cell (CSC) markers, including *ALDH1*, *CXCR4*, and *CD133*, but a much lower expression of *KRT19* than that is seen in highly aggressive triple negative breast cancer MDA-MB231 cells. Moreover, we revealed that the knockdown of *KRT19* in MDA-MB231 cells led to an enhancement of cancer properties, such as cell proliferation, sphere formation, migration, and drug resistance, while the overexpression of *KRT19* in KU-CSLCs resulted in the significant attenuation of cancer properties. *KRT19* regulated cancer stem cell reprogramming by modulating the expression of cancer stem cell markers (*ALDH1*, *CXCR4*, and *CD133*), as well as the phosphorylation of Src and GSK3β (Tyr216). Therefore, our data may imply that the modulation of *KRT19* expression could be involved in cancer stem cell reprogramming and drug sensitivity, which might have clinical implications for cancer or cancer stem cell treatment.

## 1. Introduction

Heterogeneity at the cellular and molecular level is a prominent feature of most tumor types, including breast cancer [1]. Most breast cancer cases occur in the United States of America (USA) and it is the second most prevalent cancer worldwide [2]. Diverse self-renewing cell subpopulations have been observed in primary tumors and they were shown to be genetically and phenotypically heterogeneous, possibly augmenting a number of cancer growth patterns [1]. Heterogeneity of cancer is potentially controlled by the cellular microenvironment, as well as genetic and epigenetic factors [3]. Nowadays, stem cell research becomes the hot topic to the researchers because stem cells have the ability to self-renew and differentiate into various kinds of cells [4]. Current research suggests that genetic reprogramming may play a potential role in stem/cancer stem cell biology, by sustaining the pluripotency of stem cells (SCs) and increasing their differentiation into mature cell populations [5,6]. Studies have shown that dysplastic growth could be induced by the ectopic expression of stemness marker genes in adult epithelial tissues [7,8]. In 2006, Takahashi and Yamanaka showed for the first time that pluripotent stem (PS) cells could be generated from mouse embryonic fibroblasts by ectopic expression of transcription factors (TFs), called Yamanaka factors, such as *OCT4*, *C-MYC*, *KLF4*, and *SOX2* [9]. Beside these Yamanaka factors, currently, there are around 25 TFs that are reported to be expressed in SCs. Of them, most of the TFs are suppressed in normal somatic cells, but are abnormally expressed in cancer cells [10,11], suggesting that the ectopic expression of stemness marker genes may cause the generation of abnormal cancer stem cells (CSCs). For instance, several stemness markers, such as *OCT4*, *C-MYC*, *KLF4*, *NANOG*, *SALL4*, and *SOX2* are reported to be highly expressed in various cancers and associated with poor clinical outcomes of patients [12,13,14,15,16,17,18,19,20,21,22]. Recently, another study demonstrated that early termination of reprogramming could generate cells possessing a number of stemness signatures, but was unsuccessful in transforming into induced pluripotent stem cells (iPSC) and in ending originate neoplasia, such as Wilms tumor [23]. Recent advances in stem cell biology have explored the presence of cancer stem cell-like cells (CSLCs) in several cancers, such as breast, brain, colon, leukemia, and prostate cancers [24,25,26,27,28]. CSCs are a subtype of cells that have the capacity to self-renew, produce a heterogeneous subset of cancer cells, and initiate tumor generation [29,30]. These findings may suggest a possible relationship between embryonic stem cells (ESCs)/iPSC reprogramming and tumor generation.

*KRT19* (Cytokeratin 19) is known to be the smallest (40 kDa) member of the acidic type I cytokeratin family proteins (KRTs) and may play a potential role in tumor detection by reverse transcriptase polymerase chain reaction (RT-PCR) in the bone marrow, lymph nodes, and peripheral blood of patients with breast cancer [31,32]. It has a highly preserved α-helical central domain and lack of C-terminal non-helical tail domain; α-helical central domain is very important for intermediate filament formation [33]. As a cytoplasmic intermediate filament protein, *KRT19* could be responsible for structural rigidity and multipurpose scaffolds, as well as being a marker of epithelial cells and tissues [34]. It is also known that KRTs may interact with several signal transduction molecules, such as adaptors, effectors, kinases, and receptors, which may regulate signaling pathways and mediate cell apoptosis, cell cycle arrest, invasion, and metastasis [34,35]. Recent studies have revealed that *KRT19* is crucially involved in the cancer stemness of hepatocellular carcinoma (HCC) [36]. Moreover, a recent study showed that HCC progression could be regulated through PDGFRα-laminin B1-keratin 19 cascade and this cascade could drive early recurrence, microvascular invasion, and metastasis in HCC [37,38,39]. Furthermore, it is demonstrated that *KRT19* transcription could be increased through HER2/ERK/SP1 signaling pathway, in a consequence, *KRT19* translocated to HER2 receptor then bound and stabilized the HER2 activation in breast and lung cancer [40,41]. In addition, *KRT19* is normally expressed in the stem cell region of the hair follicle [42,43], and it is overexpressed in various radio-resistant solid tumors including colon and intestine progenitor/stem cells [44]. However, *KRT19* shows discrepant relationships with both breast carcinoma and chemotherapy resistance [40,45,46,47]. Our previous study demonstrated that *KRT19* could attenuate *ALDH1*^high^/*CXCR4*^high^/*CD133*^high^ breast cancer stem cell-like cells (CSLCs) properties by regulating *NUMB*-mediated wnt/notch signaling crosstalk [46]. Moreover, *KRT19* is reported to be a tumor suppressor gene in breast cancer, which regulates the nuclear translocation of EGR1 to the *PTEN* promoter [47]. *KRT19* was also found to be a regulator of p38-MAPK/XBP-1 signaling cascade-mediated endoplasmic reticulum stress in breast cancer [48]. However, the roles that are involved in *KRT19*-driven breast cancer stem cell-like cell reprogramming and drug sensitivity are yet to be elucidated.

Herein, we have aimed to distinctly identify the role of *KRT19* in cancer stem cell reprogramming. As *KRT19* is involved in cancer regulation through signaling pathway, as we reported somewhere [46]. Here, we have hypothesized that *KRT19* may have cancer regulating role through the reprogramming of cancer stem cell by partially modulating stemness, metastasis, and drug-resistant properties. Therefore, we investigated the role of *KRT19* in cancer stem cell reprogramming and drug sensitivity by the overexpression and knockdown of *KRT19* in KU-CSLCs (konkuk university-cancer stem cell-like cell) and MDA-MB231 cells. We found that the knockdown or overexpression of *KRT19* in MDA-MB231 or KU-CSLCs cells modulated cancer reprogramming and drug sensitivity, as compared to normal MDA-MB231 or KU-CSLCs. These modulations may be partially due to the alteration of cancer stem cell markers (*ALDH1*, *CXCR4*, and *CD133*) expression as well as the phosphorylation of Src and GSK3β (Tyr216). Our result thus implies that *KRT19* has the potential for targeting highly drug-resistant CSCs and for reprogramming to less aggressive phenotypes, which may be useful for the treatment of drug-resistant breast CSCs.

## 2. Results

### 2.1. Expression of Cytokeratin 19 (KRT19) Is Downregulated in Several Breast Cancer Patients and Correlated with Breast Cancer Prognosis

**Figure 1:**
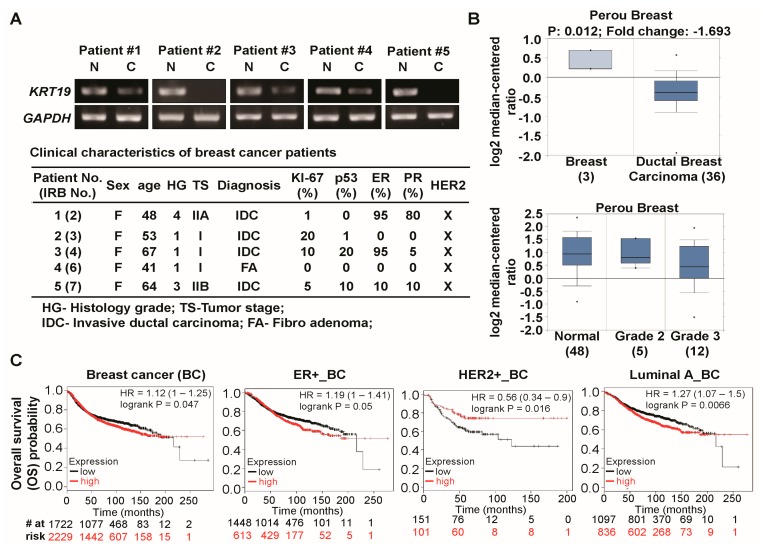
Expression pattern of *KRT19* (keratin 19) in breast cancer and its clinical significance. (**A**) mRNA expression level of *KRT19* was analyzed using reverse transcriptase polymerase chain reaction (RT-PCR) in breast cancer tissue (C; *n* = 5) as compared with adjacent normal breast tissues (N; *n* = 5). *GAPDH* was used as loading control (upper panel). Clinical characteristics of breast cancer patients for *KRT19* expression (lower panel); (**B**) Relative levels of *KRT19* expression was analyzed in normal and ductal breast carcinomas (*p* = 0.012) (upper panel) and different tumor grade of ductal breast carcinomas (lower panel) using Oncomine databases. The image was downloaded from the Oncomine datasets provided by Perou et al. [49]; (**C**) Kaplan–Meier curves for overall survival (OS) probability of patients with breast cancer (BC) (*n* = 3951); ER+_BC (*n* = 2061); HER2+_BC (*n* = 252); and Luminal A_BC (*n* = 1933) expressing high and low expression levels of *KRT19*.

As *KRT19* expressed in various cancer types [36,37,38,39,40,41,44,45,46,47], we checked the expression level of *KRT19* in five paired breast cancer tissue and adjacent normal breast tissues using reverse transcriptase-polymerase chain reaction (RT-PCR) analysis. However, our RT-PCR results showed that the expression of *KRT19* was attenuated in breast cancer tissue (C) when compared to their normal (N) counterparts (Figure 1A, upper panel); the clinical characterization of breast cancer patients was overviewed in Figure 1A, lower panel. We then checked the *KRT19* expression in breast cancer using Oncomine database. The *KRT19* was significantly downregulated in ductal breast cancer tissue as compared to the normal tissue (Figure 1B, upper panel) [49], although *KRT19* expression was upregulated in other breast cancer studies (Oncomine). Moreover, *KRT19* expression downregulated in more aggressive breast cancer tissue than normal and less aggressive tissue (Figure 1B, lower panel) [49]. Next, we aimed to investigate the relationship between *KRT19* expression and the patient’s clinical outcomes using the Kaplan-Meier plotter. We found that patients with low expression of *KRT19* were significantly correlated with poor prognosis in breast cancer (BC), ER+_, HER2+_, and luminal A_BC patients (Figure 1C), suggesting that *KRT19* is downregulated in breast cancer and is significantly correlated with poor breast cancer prognosis.

### 2.2. Chemo-Treated Breast Cancer Patient-Derived KU-CSLCs Show High Expression of ALDH1, CXCR4, and CD133, and More Aggressive Cancer Phenotypes

**Figure 2:**
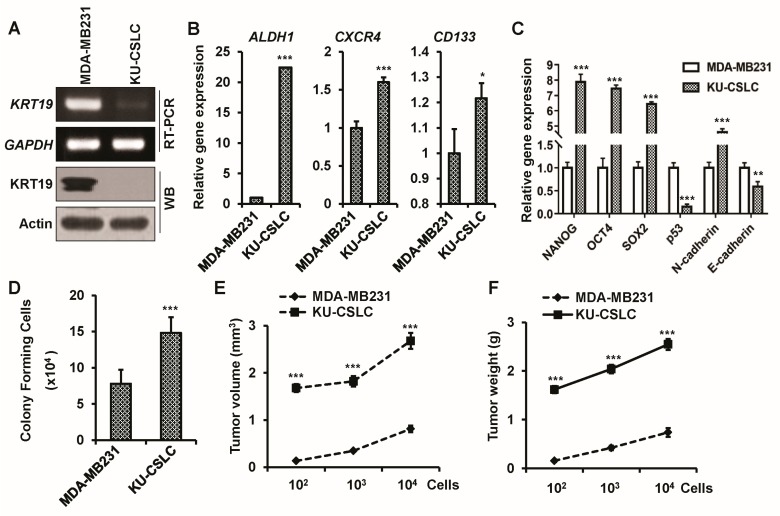
Patient-derived cancer stem-like cells (konkuk university-cancer stem cell-like cell (KU-CSLCs)) show downregulated *KRT19* expression and highly aggressive cancer stem cell-like cells (CSLC) properties. (**A**) mRNA and protein expression levels of *KRT19* were analyzed using RT-PCR and western blot (WB) in MDA-MB231 and KU-CSLC cells; (**B**) mRNA expression levels of *ALDH1*, *CXCR4*, and *CD133* were analyzed using real-time polymerase chain reaction (qRT-PCR) in MDA-MB231 and KU-CSLC cells. The specific band intensity was measured by the scanning densitometry program in ImageJ and normalized to that of a housekeeping gene, *GAPDH*. * *p* < 0.05; *** *p* < 0.001; (**C**) mRNA expression levels of stemness (*NANOG, OCT4*, and *SOX2*), epithelial-to-mesenchymal (EMT; *N-* and *E-cadherin*) markers, and *P53* were analyzed using qRT-PCR in MDA-MB231 and KU-CSLC cells. ** *p* < 0.01; *** *p* < 0.001; (**D**) Colony formation assay was analyzed in serum-free media (SFM) using non-coated plates. After five days of culture, colonies from either KU-CSLCs or MDA-MB-231 cells were collected and stained with crystal violet. The stained colonies were enumerated and presented as colony forming cells (×10^4^). *** *p* < 0.001; (**E**,**F**) An indicated number of MDA-MB231 or KU-CSLC cells were injected into SCID mice (*n* = 5) and after four weeks of post tumor formation, the mice were excised. Tumor volume (mm^3^) and weight (g) were assessed after the biopsy. *** *p* < 0.001.

It is known that the overexpression of *ALDH1*, *CXCR4*, and *CD133* produces cells with an aggressive and highly drug-resistant cancer stem cell-like phenotype [50,51]. Our previous study reported that the expression of *KRT19* was upregulated in various breast cancer cell lines (i.e., MDA-MB231, MCF7) and was downregulated in highly aggressive cancer stem-like cells [46]. In our present study, we have also confirmed the expression pattern of *KRT19* in MDA-MB231 and highly aggressive and drug-resistant breast cancer patient-derived cancer stem cell-like KU-CSLC (konkuk University-cancer stem cell-like cell) cells, using RT-PCR and western blot (WB) analysis (Figure 2A). To characterize chemo-treated breast cancer patient-derived KU-CSLCs, a triple negative breast cancer cell line, MDA-MB231, and KU-CSLC cells, were analyzed for the expression of *ALDH1*, *CXCR4*, and *CD133* while using RT-PCR. The data indicated that the expression of *ALDH1*, *CXCR4*, and *CD133* was significantly upregulated in KU-CSLC cells (Figure 2B). We then checked the expression of stemness marker genes (*NANOG*, *OCT4*, and *SOX2*), EMT markers (*N-cadherin* and *E-cadherin*), and the tumor suppressor gene *P53*, using qRT-PCR analysis. The KU-CSLC cells showed a significant upregulation of stemness marker genes (*NANOG*, *OCT4*, and *SOX2*) and *N-cadherin*, while a downregulation pattern of *E-cadherin* and *P53* was observed in KU-CSLC cells when compared to MDA-MB231 cells (Figure 2C). The colony formation efficiency was significantly higher in KU-CSLC cells as compared to MDA-MB231 cells (Figure 2D). Afterward, the in vivo tumorigenic efficiency was investigated for both MDA-MB231 and KU-CSLCs. To verify in vivo tumorigenic efficiency, we used five-month old female SCID mice and subcutaneously implanted a range of cell numbers, 100, 1000, and 10,000, of both MDA-MB231 and KU-CSLC cells into the lower flanks of the mice. The tumor generation and growth in mice were monitored and mice were sacrificed four weeks post tumor formation. The results showed that for KU-CSLC cells, even 100 cells were enough to significantly induce tumor growth when compared to MDA-MB231 cells, which required 10,000 cells (Figure 2E). Concurrently, tumor weights from the KU-CSLC cells were significantly heavier than those resulting from MDA-MB231 cells (Figure 2F). These results suggest that chemo-treated breast cancer patient-derived KU-CSLC cells are more aggressive and drug-resistant than the aggressive triple negative MDA-MB231 cells. 

### 2.3. Knockdown or Overexpression of KRT19 Regulates Cell Proliferation and Sphere Formation in MDA-MB231 and KU-CSLC Cells

**Figure 3:**
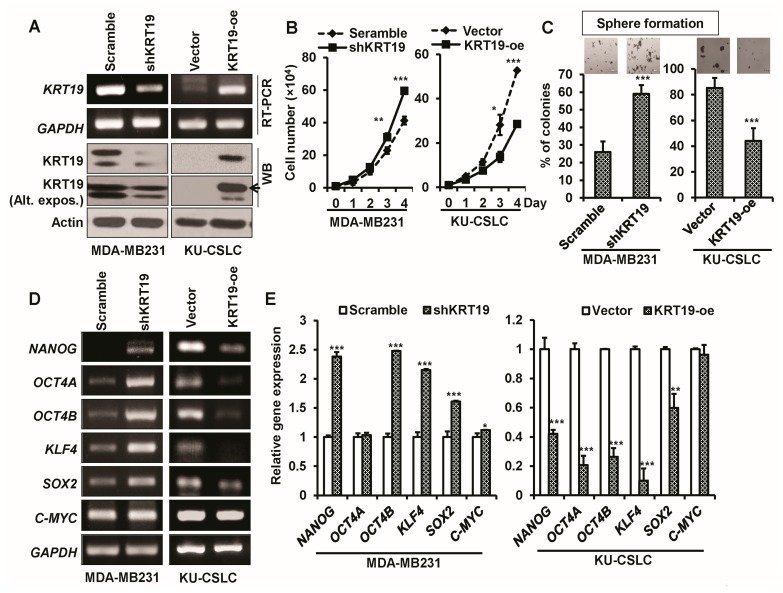
*KRT19* could regulate cancer properties in MDA-MB231 or KU-CSLC cells. (**A**) mRNA and protein expression levels of *KRT19* were analyzed using RT-PCR and WB in the MDA-MB231-scramble, MDA-MB231-shKRT19, KU-CSLC-vector, and KU-CSLC-KRT19-oe (keratin19-overexpression) cells, respectively. Alternative exposure (Alt. expos.) was used for *KRT19* protein expression in indicated cells; (**B**) The effects of *KRT19*-silencing or -overexpression on cell proliferation were analyzed by cell counting. Cells were enumerated up to four days in the indicated virus-infected cells. * *p* < 0.05; ** *p* < 0.01; *** *p* < 0.001. (**C**) Cells were cultured in sphere-forming media (SFM) using non-coated plates. The spheres were stained in indicated cells by crystal violet and were counted on day five. The spheres of the crystal violet stained cells were captured by phase contrast microscopy and presented as a percentage (%) of colonies. *** *p* < 0.001. (**D**,**E**) mRNA expression levels of stemness marker genes (*NANOG*, *OCT4A*, *OCT4B*, *KLF4*, *SOX2*, and *C-MYC*) were analyzed in the indicated cells using RT-PCR analysis. The specific band intensity was measured by the scanning densitometry program in ImageJ and normalized to that of a housekeeping gene, *GAPDH*. * *p* < 0.05; ** *p* < 0.01; *** *p* < 0.001.

As *KRT19* was upregulated in MDA-MB231 and downregulated in KU-CSLC cells, we next knocked down the expression of *KRT19* by using a *KRT19* specific shRNA (shKRT19) and overexpressed *KRT19* (KRT19-oe) in KU-CSLC cells to investigate the function of *KRT19* in breast cancer. The knockdown and overexpression of *KRT19* were confirmed by RT-PCR and WB analysis (Figure 3A). Cell proliferation analysis demonstrated that cell proliferation was increased in shKRT19 infected MDA-MB231 cells and decreased in the *KRT19* overexpressing KU-CSLC cells, when compared to their control counterparts (Figure 3B). To further explore the role of *KRT19* on cell growth, we investigated the sphere formation ability using a sphere-forming assay. We observed a significantly increased number of the spheres in MDA-MB231 cells that were infected with the shKRT19 virus, as compared to the scramble, whereas a reduced sphere forming ability was seen in *KRT19* overexpressing KU-CSLC cells, when compared with those infected with the vector (Figure 3C). A subsequent RT-PCR analysis for stemness marker genes was performed, with the results showing that the expression of stemness markers (*NANOG*, *OCT4B*, *KLF4*, *SOX2*, and *C-MYC*) was significantly increased in shKRT19 virus-infected MDA-MB231 cells and downregulated in *KRT19* overexpressing KU-CSLC cells, as compared to scramble or vector infected MDA-MB231 or KU-CSLC cells, respectively (Figure 3D,E), suggesting that *KRT19* may regulate cell growth and sphere formation ability in breast cancer.

### 2.4. Silencing or Upregulation of KRT19 Regulates Cell Migration in MDA-MB231 and KU-CSLC Cells

**Figure 4:**
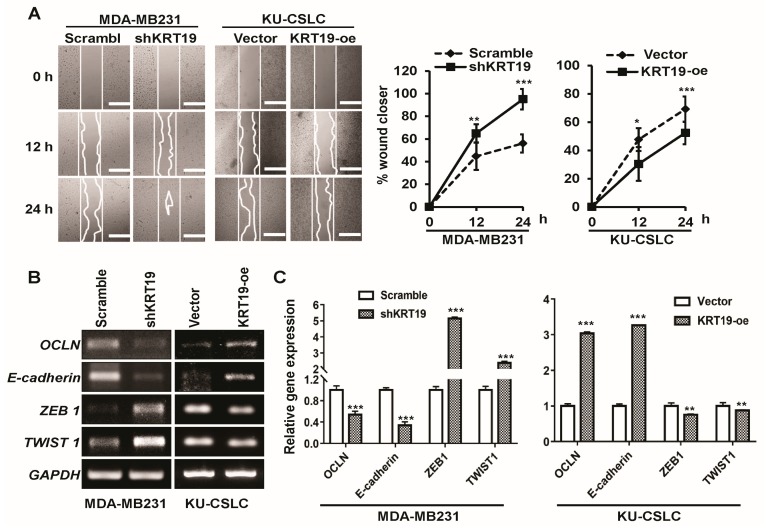
Knockdown or overexpression of *KRT19* highly enhances or inhibits cancer wound healing migration ability in MDA-MB231 or KU-CSLC cells. (**A**) Migration ability of the indicated cells was analyzed using a wound-healing migration assay. The percentage (%) of the enclosure was captured by phase contrast microscopy (left panel) (Scale bar represents 500 μm) and was presented as a percentage (%) of the enclosure (right panel) at the indicated time points. * *p* < 0.05; ** *p* < 0.01; *** *p* < 0.001; (**B**,**C**) mRNA expression levels of epithelial and mesenchymal transition (EMT) marker genes (*OCLN*, *E-cadherin*, *ZEB1*, and *TWIST1*) were analyzed in the indicated cells using RT-PCR analysis. The specific band intensity was measured using the scanning densitometry program in ImageJ and normalized to that of a housekeeping gene, *GAPDH*. ** *p* < 0.01; *** *p* < 0.001.

To explore the function of *KRT19* on cell migration in breast cancer, we first performed a wound healing cell migration analysis. The results showed that cell migration during wound healing was increased upon *KRT19* knockdown in MDA-MB231 cells, while a reduction in cell migration was observed in *KRT19*-overexpressing KU-CSLC cells, when compared to those of scramble- or vector-expressing MDA-MB231 or KU-CSLC cells, respectively (Figure 4A). Afterwards, an RT-PCR analysis was performed to assess the expression of EMT marker genes, with the results displaying that MET (mesenchymal to epithelial transition) markers (*OCLN* and *E-cadherin*) were downregulated and EMT markers (*ZEB1* and *TWIST1*) were upregulated in *KRT19*-silencing MDA-MB231 cells, whereas the opposite result was observed in *KRT19*-overexpressing KU-CSLC cells as compared to their control precursors (Figure 4B,C). This suggested that the silencing or overexpression of *KRT19* could regulate cell migration in MDA-MB231 or KU-CSLC cells.

### 2.5. KRT19 Can Regulate Drug Resistance Capacity in Breast Cancer Cells

**Figure 5:**
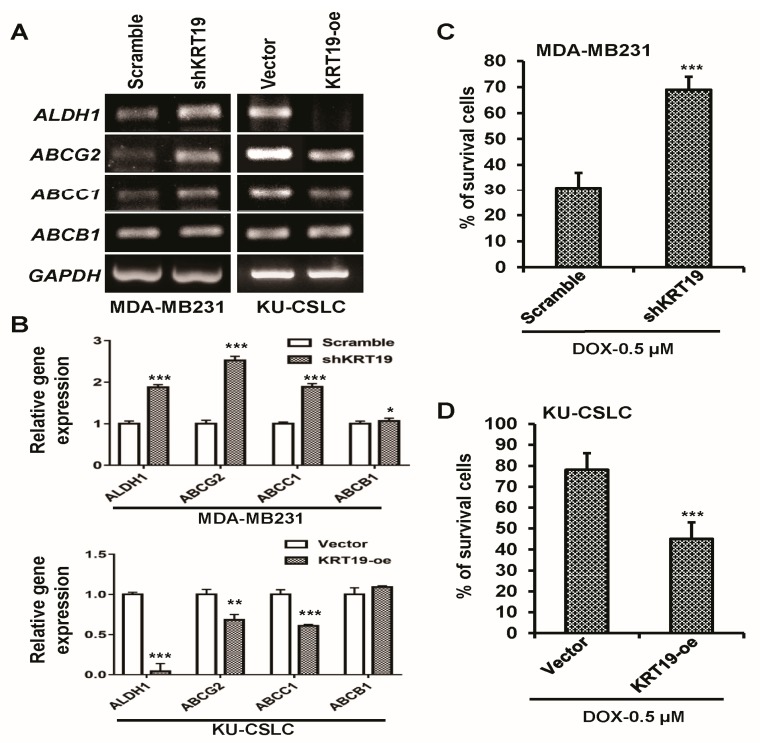
Silencing or overexpression of *KRT19* strongly induces or attenuates cancer drug-resistance capacity in MDA-MB231 or KU-CSLC cells. (**A**,**B**) mRNA expression levels of multidrug-resistant (MDR) marker genes (*ALDH1*, *ABCG2*, *ABCC1*, and *ABCB1*) were analyzed in the indicated cells using RT-PCR analysis. The specific band intensity was measured using the scanning densitometry program in ImageJ and normalized to that of a housekeeping gene, *GAPDH*. * *p* < 0.05; ** *p* < 0.01; *** *p* < 0.001; (**C**,**D**) The effects of *KRT19* silencing or overexpression on drug resistance was quantified by cell enumeration after 48 h of doxorubicin treatment (0.5 µM) in the indicated cells. Data are shown as percentages (%) of survived cells. *** *p* < 0.001.

To further examine the function of *KRT19* on drug sensitivity, we examined the expression of drug-resistance marker genes using RT-PCR analysis. The results showed that the knockdown of *KRT19* significantly upregulated the expression of drug-resistance markers (*ALDH1*, *ABCG2*, *ABCC1*, and *ABCB1*), as compared to those of the control shRNA-infected MDA-MB231 cells (Figure 5A,B). We then checked the cell survival in the presence of several doses of doxorubicin (DOX), which is a well-known anti-cancer drug, in both MDA-MB231 and KU-CSLC cells, to assess drug resistance capability (Supplementary Figure S1). A specific dose (0.5 µM) was chosen for further study. The cell survival results in the presence of DOX (0.5 µM) showed that silencing of *KRT19* significantly augmented cell survival, compared to those of scramble-infected MDA-MB231 cells (Figure 5C). We then assessed the expression of drug-resistance marker genes during *KRT19* overexpression in KU-CSLCs. The RT-PCR analysis of drug-resistance markers revealed a significant downregulation in *KRT19* overexpressing KU-CSLCs, when compared to their normal counterparts (Figure 5A,B). Subsequently, we checked drug-resistance capability in the presence of DOX, by overexpressing *KRT19* in highly aggressive drug-resistant KU-CSLC cells. To determine drug-resistance capabilities, cell survival analysis was performed in the presence of DOX (0.5 µM) in both vector- and *KRT19*-overexpressing KU-CSLC cells. Cell survival was significantly suppressed in *KRT19*-overexpressing cells as compared to those of vector-infected KU-CSLC cells (Figure 5C), implying that *KRT19* may regulate drug-sensitivity in highly drug-resistant breast cancer stem cell-like cells.

### 2.6. KRT19 Regulates Cancer Stem Cell Reprogramming through p-GSK3β (Tyr216) and p-Src Signaling Pathway

**Figure 6:**
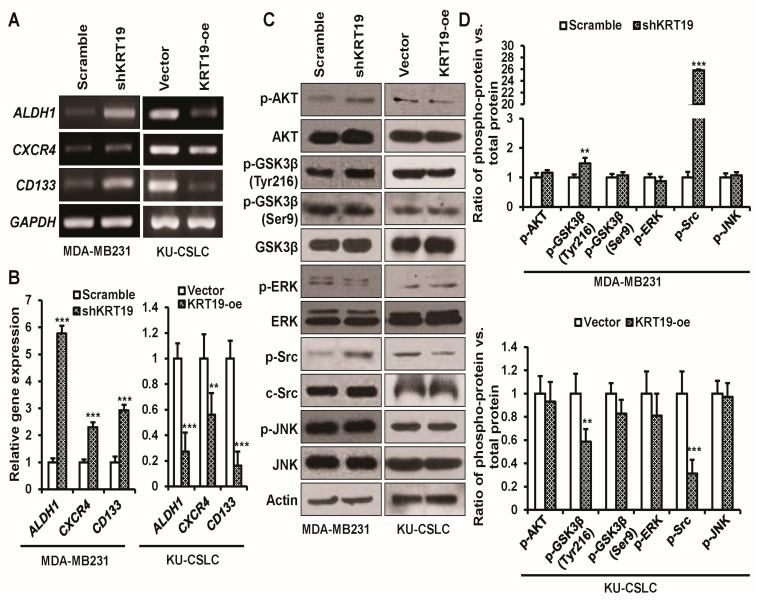
*KRT19* regulates cancer stem cell reprogramming through p-GSK3β (Tyr216) and p-Src signaling pathway. (**A**,**B**) mRNA expression levels of cancer stem cell markers (*ALDH1*, *CXCR4*, and *CD133*) were analyzed in the indicated cells while using RT-PCR analysis. The specific band intensity was measured using the scanning densitometry program in ImageJ and normalized to that of a housekeeping gene, *GAPDH*. ** *p* < 0.01; *** *p* < 0.001; (**C**,**D**) Representative WB analysis of the AKT, GSK3β, ERK, Src, and JNK signaling proteins in indicated cells. The specific band intensity was measured by the scanning densitometry program in ImageJ and normalized to that of a total protein. ** *p* < 0.01; *** *p* < 0.001.

As the KU-CSLC showed high *ALDH1*, *CXCR4*, and *CD133* gene expression, and low *KRT19* expression, we assessed the effect of *KRT19* overexpression or knockdown in KU-CSLC or MDA-MB231 cells. The RT-PCR analysis revealed that the overexpression or knockdown of *KRT19* could modulate *ALDH1*, *CXCR4*, and *CD133* gene expression in KU-CSLC or MDA-MB231 cells (Figure 6A,B). We next aimed to explore the underlying mechanism by which *KRT19* regulates cancer reprogramming. Although *KRT19* regulates EGR1/PTEN/AKT, β-catenin/NUMB/NOTCH, and HER2/ERK/SP1 cascades [29,46,47], we focused on the AKT, GSK3β, ERK, Src, and JNK signaling pathway in this study, as these signaling pathways are crucially involved in cancer progression [52,53,54,55,56]. The WB analysis demonstrated that the phosphorylation of p-GSK3β (Tyr216) and p-Src levels were increased significantly upon the knockdown or overexpression of *KRT19* in MDA-MB231 or KU-CSLC cells, while the expression of p-AKT, p-GSK3β (Ser9), p-ERK, and p-JNK was unchanged (Figure 6C,D), which is suggested that *KRT19* could regulate the cancer stem cell reprogramming by partially modulating the phosphorylation of GSK3β (Tyr216) and Src in breast cancer or cancer stem cells.

## 3. Discussion

CSCs (cancer stem cells) share several characteristics with typical stem cells, such as self-renewal and their undifferentiated and differentiated states. CSCs have been reported to originate from cancer cells with non–stemness properties, via a reprogramming mechanism that is very similar to that which is involved in iPSC (induced pluripotent stem cells) generation [23]. In addition, several key transcription factors, including *NANOG*, *OCT4*, *KLF4*, and *SOX2*, which induced iPSC and maintained the stemness properties of embryonic and somatic stem cells, have also been highly expressed in CSCs [8,57,58]. A number of recent studies have reported that cancer could be developed by the early termination of the reprogramming process in numerous tissues, through altered genetic and epigenetic regulation [7,8,23]. Thus, it could be inferred that teratoma formation from iPSCs is strongly associated with premature reprogramming, which is also important for the development of CSCs. In our current study, we have reported a relationship between *KRT19* (cytokeratin 19) expression with CSC reprogramming, and drug sensitivity in breast cancer and cancer stem cell-like cells. Moreover, we discovered that *KRT19* regulates CSC reprogramming and drug sensitivity in breast cancer and cancer stem cell-like cells, through mediating stemness (*NANOG*, *OCT4*, *KLF4*, and *SOX2*) and drug-resistant (*ALDH1*, *ABCG2*, *ABCC1*, and *ABCB1*) marker expression. Concurrently, we showed that *KRT19* regulates breast cancer cell migration and EMT (epithelial to mesenchymal transition)-responsive gene expression. However, further study may elucidate the function of *KRT19* in key regulatory pathways upon cancer reprogramming and drug sensitivity in various cancer stem cells. 

*KRT19*, a cytokeratin protein, is reported to be differentially expressed in several cancers. It is highly expressed in breast, colon, liver, and intestine cancer, and it is associated with poor clinical outcomes in patients [36,40,44,45]. *KRT19* expression, however, is also known to be negatively correlated with cancer progression in breast cancer stem cell-like cells and cell lines [46,47,48]. So far, the function of *KRT19* in breast cancer still remains to be revealed. In this study, we found the downregulated *KRT19* expression pattern in breast cancer tissue when compared to the adjacent normal tissue, which is an agreement with Oncomine database. Oncomine search, a freely accessible database, could make the research easier to correlate the experimental data with published genome-wide RNAseq data [59,60]. The *KRT19* expression is reported to be correlated with cancer progression and prognosis [36,44,45,46,47]. Our data also evidences that *KRT19* expression is significantly associated with clinical outcomes in breast cancer. In this current study, we also used patient-derived KU-CSLC cells, which have high drug-resistance, stemness, tumorigenic, and aggressive cancer stem cell-like cell properties, and a commercially available aggressive MDA-MB231 cell line to identify the role of *KRT19* in cancer reprogramming and drug sensitivity. Our results also demonstrated that the expression of *KRT19* was very low in highly aggressive KU-CSLC cells, when compared to those of MDA-MB231, which we previously reported on [46]. In this study, we further demonstrated that the knockdown of *KRT19* in MDA-MB231 cells significantly induced cell proliferation and sphere formation, suggesting that *KRT19* is a potent cancer suppressor gene. Moreover, the silencing of *KRT19* augmented cell migration and drug-resistance of MDA-MB231, which means that the low expression of *KRT19* produced an aggressive phenotype in cancer cells, as previously reported [40,46,47].

In addition, we demonstrated that the expression of *ALDH1, CXCR4,* and *CD133* was higher in KU-CSLCs than in the aggressive MDA-MB231 cell line. It is also reported that *ALDH1, CXCR4,* and *CD133* are well-known markers of highly aggressive, drug-resistant, invasive, and cancer stem cell phenotypes [50,51], suggesting that our KU-CSLCs are highly aggressive and drug-resistant cancer stem cell-like cells. Recent studies indicate that the aberrant expression of *ALDH1* contributes to cancer stem cell transformation from less aggressive cancer with the association of *CD133* [61,62]. Moreover, *CD133*+ and *CXCR4*+ are reported to involve in cancer stem cell phenotype in various cancer types [63,64,65]. Thus, targeting *ALDH1*, CRCR4, and *CD133* could provide us a possibility to target cancer stem cell population. We have demonstrated that *KRT19* overexpression inhibited aggressiveness, including abnormal cell proliferation, sphere formation, migration, and drug resistance in KU-CSLCs. Moreover, key transcription factors comprising *OCT4*, *KLF4*, *NANOG*, and *SOX2*, which enhanced the iPSC generation and maintained the self-renewal properties in embryonic and somatic stem cells, have also been highly expressed in CSCs [8,57,58]. Previous studies also demonstrated that these transcription factors involved in cancer progression and associated with clinical outcomes [12,13,14,15,16,17,20,21,22]. Our data showed a corroborative evidence that overexpression/knockdown of *KRT19* significantly altered the expression of *OCT4*, *KLF4*, *NANOG*, and *SOX2* in KU-CSLC or MDA-MB231 cells. This implies that through *OCT4*, *KLF4*, *NANOG*, and *SOX2* alteration, *KRT19* regulates cancer stem cell reprogramming efficiency in KU-CSLCs.

Cancer metastasis is one of the crucial factors to develop cancer stem cell phenotype [66]. It is noted that stemness markers expression also regulates the metastasis properties in cancer cells [67,68,69,70]. Targeting cancer stem cell metastasis through the regulation of stemness markers would be the worthy solution in cancer treatment. Moreover, it is reported that *OCT4* expression mediates cancer stem cell reprogramming in *CD133*+ lung cancer cells [71]. It also mediated drug resistance in prostate cancer. Other stem cell markers, in addition, contributed to cancer stem cell recurrence and drug resistance [72,73,74,75]. Our confirmative data showed that overexpression/knockdown of *KRT19* significantly regulated the expression of EMT [76] and drug-resistance [77] markers in both KU-CSLC or MDA-MB231 cells, which may partially due to the regulation of stemness markers expression and imply a partial regulation of cancer phenotypes and sensitivity to potent anti-cancer drugs.

**Figure 7:**
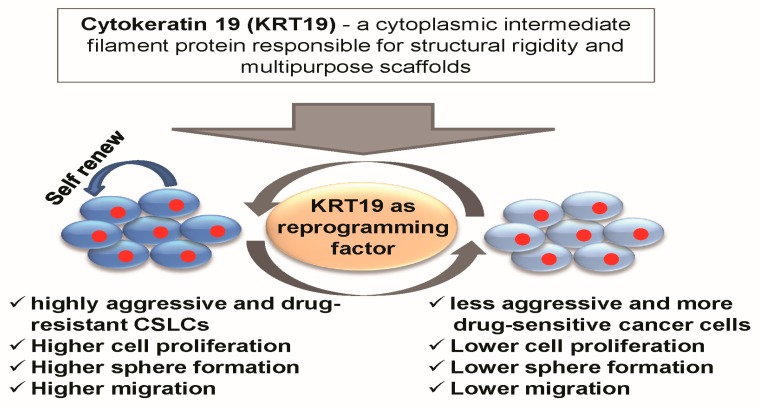
Schematic diagram demonstrating the *KRT19*-mediated modulation of cancer reprogramming.

The pathologic reprogramming procedure in cancer is reported to be critical in regulating the CSC induction and cancer reappearance [78,79]. In our data, *KRT19* not only regulates cancer stemness, metastasis, and drug resistance, but it also modulates the expression of stemness, metastatic, and drug-resistant markers. This phenomenon could mediate through the signaling pathways. It is reported that *KRT19* regulates several signaling cascades, including PDGFRα/laminin B1/keratin 19, HER2/ERK/SP1, WNT/NUMB/NOTCH, EGR1/PTEN/AKT, etc. [37,40,41,46,47]. In this study, we have reported that *KRT19* regulated the phosphorylation of Src and GSK3β at Tyr216, while the other signaling proteins, such as p-AKT, p-ERK, and p-JNK were unregulated. Src is reported to support *ALDH1*+ CSC phenotype and reprogramming in various cancer types, including breast cancer [80]. Moreover, Src is also involved in the regulation of invasion and migration in several cancer types [81,82] and the ablation of Src could alternate the CSC phenotype [83], which is corroborated with our data. Previous studies also reported that dysregulation of Src and GSK3β signaling could mediate cancer progression, metastasis, drug resistance, and ultimately transform into cancer stem cell phenotypes [83,84,85,86]. Factually, alterations in GSK-3 activity have been explored due to the changes in GSK-3α and GSK-3β phosphorylation at Ser21 and Ser9, respectively, by Akt activation [87]. But, the activation of GSK-3α and GSK-3β depends on the phosphorylation at Tyr-279 and Tyr-216 [88,89]. Moreover, a recent in vitro and in vivo study showed that targeting Src-mediated GSK3β phosphorylation at Tyr216 could inhibit prostate cancer progression [86]. Src is also essential for the wound healing and fibrotic events where p-GSK3β (Tyr216) played an important role in fibrotic events mean that Src kinase may modulate the GSK3β activity [86], which might be in agreement with our study that *KRT19* mediated cancer stem cell reprogramming might be through the regulation of Src/GSK3β phosphorylation at Tyr216 in breast cancer. Hence, the function of *KRT19* in cancer stem cell reprogramming and the drug-resistance pathway is remarkable (Figure 7), and it would be interesting to explore the underlying mechanisms in detail.

## 4. Materials and Methods

### 4.1. Human Tissue Samples

Human breast tumor and normal tissue samples were collected from the Breast Cancer Center of Konkuk University Hospital with Institutional Review Board (IRB, KUH 1020003) approval. Breast tumor and normal tissues were collected from neoadjuvant chemotherapy (four cycles of preoperative Doxorubicin and cyclophosphamide)-treated stage III patients. The occupied tissues were rinsed with PBS (phosphate-buffered saline) and were incubated with type IV collagenase (Sigma-Aldrich, St. Louis, MO, USA) for 20 min at room temperature (RT). A single-cell suspension was prepared by filtering the supernatant through a 100 μm cell strainer (BD Bioscience, San Jose, CA, USA) and was cultured in DMEM (Dulbecco’s Modified Eagle’s medium; Sigma-Aldrich, Saint Louis, MO, USA) supplemented with 10% FBS (fetal bovine serum; GE Healthcare Hyclone, Pittsburgh, PA, USA) and 1% P/S (100 U/mL penicillin/streptomycin; GE Healthcare Hyclone) at 37 °C with 5% CO_2_. Unless stated otherwise, cells were cultured on the adherent tissue culture plates (Nunc). Cancer (C) and normal (N) tissue samples from five patients were used in the study. 

### 4.2. Bioinformatics Analysis

Oncomine database (www.oncomine.org/) was used to obtain the *KRT19* expression in breast cancer tissue and their normal tissues [59,60]. The fold change of *KRT19* mRNA expression in breast cancer tissue when compared to their normal counterparts was obtained as the parameters of threshold *p*-value: 1 × 10^−4^; fold change: 2; and, gene ranking in the top 10%.

Kaplan–Meier curves were obtained using the KMplot program (http://kmplot.com/analysis/) [90]. To analyze the clinical outcomes of *KRT19* gene, the patient samples were split into two groups, according to the median gene expression of the probe of *KRT19* (201650_at). The log-rank *p*-value and the hazard ratio with 95% confidence intervals was also calculated. 

### 4.3. Cell Culture

MDA-MB231, a human breast cancer cell line, was acquired from ATCC (American Type Culture Collection, Manassas, VA, USA). KU-CSLCs (konkuk University-cancer stem cell-like cell; *CD133*^high^/*CXCR4*^high^/*ALDH1*^high^) were collected from breast tumor tissue specimens, which were derived from chemo-treated patients at the Breast Cancer Center, Konkuk University Hospital, Seoul. The study was performed with the consent of the Institutional Review Board (IRB, KUH 1020003). Cells (MDA-MB231 and KU-CSLCs) were cultured in either RPMI (Roswell Park Memorial Institute) 1640 or high glucose-DMEM (Sigma-Aldrich) that was supplemented with 10% FBS (Hyclone) and 1% penicillin/streptomycin (Hyclone) at 5% CO_2_ and 37 °C in a humidified incubator. Short tandem repeat (STR) profiling was used for cell authentication and cells were tested by BioMycoX*^®^* Mycoplasma PCR Detection Kit for mycoplasma contamination detection (Cat. No. CS-D-25) (Cellsafe, Yeongtong-Gu, Suwon, Republic of Korea).

### 4.4. Total RNA Extraction and Reverse Transcriptase-PCR (RT-PCR) Analyses 

For total RNA isolation, cells were harvested using a total RNA extraction kit (iNtRON Biotechnology, Jungwon-Gu, Seongnam-Si, Gyeonggi-do, Republic of Korea) by following the manufacturer’s instructions. The concentration of total RNA was quantified using a Nanodrop (ND1000) (Nanodrop Technologies Inc., Wilmington, DE, USA). Afterward, cDNA was synthesized using 1–2 μg of total RNA with M-MLV reverse transcriptase (RTase) (Promega, Madison, WI, USA), following the manufacturer’s instructions. 

### 4.5. Polymerase Chain Reaction (PCR) and Quantitative Real-Time-PCR (qRT-PCR) 

After the cDNA synthesis, PCR analysis was carried out using rTaq Plus 5X PCR master mix (Elpis biotech, Seo-gu, Daejeon, Republic of Korea), following the manufacturer’s instructions. The primer sequences that were used in this study are listed in Table 1. The PCR products were run on 1.5% agarose gels and imaged. The density of the PCR band was analyzed using ImageJ software. Quantitative real-time-PCR was performed using a thermal cycler (PTC-200) with a Chromo4 optical detector (MJ Research/BioRad, Hercules, CA, USA) using Fast 2X SYBR Green Master Mix (Applied Biosystems, Stockholm, Sweden). The housekeeping gene *GAPDH* was used as loading control, as described previously [46,91].

### 4.6. In Vivo Tumorigenicity Analysis

For in vivo tumorigenicity analysis, five-month old female SCID (severe combined immunodeficiency) mice were used [92], which were obtained from Korean Research Institute of Bioscience and Biotechnology (KRIBB) (Republic of Korea). For generating the tumor, MDA-MB231 or KU-CSLCs cells (10^2^, 10^3^, or 10^4^ cells) were inoculated subcutaneously into the lower flanks of the mice. The tumor formation and growth of the mice were monitored, and the mice were sacrificed at four weeks post-tumor generation. Subsequent tumor volumes and weights were calculated, as described previously [93]. During the study, mice were allocated into two groups, non-randomly, based on their body weight. Each group contained at least five mice. The mice were omitted if they died or did not generate any tumor. The investigator was blinded during the study for the allocation of the group. According to the institutional guidelines, the mice were cared for and treated with permission (KU16003).

### 4.7. Knockdown and Overexpression of KRT19 

For short hairpin RNA (shRNA) knockdown assay, sense and antisense oligonucleotides for control (scramble), and *KRT19* (shKRT19), as described in our previous report [46], containing BamHI and EcoRI restriction sites, were annealed using annealing buffer [100 mM NaCl, Tris-EDTA (TE) buffer (pH 8) (Sigma-Aldrich)]. Afterward, the annealed double-stranded oligonucleotides were cloned into the pGreenPuro lentiviral shRNA expression vector (System Biosciences, Mountain View, CA, USA) to construct shKRT19 or Scramble vectors. 

For the overexpression of *KRT19*, first, its complete coding sequence (CDS) was cloned into the pGEM^®^-T Easy vector (Promega) using the T vector primers, which were designed by the primer3 program (http://primer3.ut.ee/). The complete CDS without the stop codon was further cloned into the pCDH-EF1-MCS-T2A-copGFP (pCDH) lentiviral vector (System Biosciences) containing XbaI and EcoRI restriction sites, to construct the KRT19-oe vector, with the empty pCDH vector used as a control (vector). The primers that were used for the T Easy vector and pCDH vector clone were described in our previous report [46]. 

For lentivirus production, human HEK293T cells were transfected with viral packaging plasmids (i.e., RRE and REV) and the specific lentiviral expression vector using the calcium phosphate standard transfection protocol. Viruses were collected and were concentrated using an ultracentrifuge (Optima L-90K, Beckman Coulter Inc., Brea, CA, USA) at 48 h post incubation, at 5% CO_2_ and 37 °C. Either scrambled or shKRT19 lentiviruses were then infected to MDA-MB231 cells, while KU-CSLCs were infected with either control (vector) or *KRT19*-overexpressing (KRT19-oe) lentiviruses and were incubated overnight at 5% CO_2_ and 37 °C. Afterward, the virus-containing media was changed and cells were maintained with normal growth media. To produce stably expressing cells, ~8.0 × 10^8^ IU/mL viral particles were incubated with cells. To calculate virus titer, we used the following equation, titer in IU/mL = [(number cells at starting time) × (dilution factor) × (percent of infection)/(volume of virus solution added, expressed in mL)], as described previously [94]. The <95% virus-infected GFP-expressing cells were used for the future study, and knockdown or overexpression was confirmed by RT-PCR and WB analyses.

### 4.8. Western Blotting (WB) Analysis

For WB analysis, cells were harvested using cell lysis buffer [100 mM Tris-HCl (pH 7.5), 1% Triton X-100 (Sigma-Aldrich), 10 mM NaCl, 10% glycerol (Amresco, Solon, OH, USA), 50 mM sodium fluoride (Sigma-Aldrich), 1 mM sodium orthovanadate (Sigma-Aldrich), 1 mM phenylmethylsulfonyl fluoride (PMSF; Sigma-Aldrich), and 1 mM *p*-nitrophenyl phosphate (Sigma-Aldrich)], and the lysates were centrifuged at 13,000 rpm for 15 min at 4 °C. For protein quantification, Bradford protein assay reagent (BioRad) was used, and the quantified proteins were separated either 10 or 12% sodium dodecyl sulfate-polyacrylamide gel electrophoresis (SDS-PAGE). The separated proteins were transferred onto nitrocellulose membranes (Bio-Rad, Philadelphia, PA, USA). Afterward, the membranes were stained with 1× Ponceau staining for 5 min, and then blocked with 5% skimmed milk in Tris-buffered saline (TBS) for 1 h. The membranes were then incubated with primary antibodies against *KRT19* (SC-53258), AKT (SC-1619), phospho (p)-AKT (SC-16646-R), GSK3β (SC-9166), p-GSK3β-Ser9 (SC-11757), ERK (SC-153), p-ERK (SC-7383), c-Src (SC-18), JNK (SC-7345), p-JNK (SC-6254), actin (SC-1616) (Santa Cruz Biotechnology, Dallas, TX, USA) p-Src (2101S) (Cell signaling, Danvers, MA, USA), and p-GSK3β-Tyr216 (05-413) (Merck Millipore, Burlington, MA, USA), overnight at 4 °C on a shaker. After overnight incubation, the membranes were washed with TBST for 30 min and were then incubated with appropriate second antibodies, including anti-mouse (SC-2005), -goat (SC-2020), or -rabbit (SC-2004) IgGs tagged with horseradish peroxidase (HRP) for 2 h at room temperature (Santa Cruz Biotechnology). After washing the membranes, protein expressions were detected on X-ray films using an enhanced chemiluminescence (ECL) kit (Amersham Bioscience, Piscataway, NJ, USA), as described previously [46,91,95]. To detect the expression of *KRT19*, we exposed the X-ray films for alternative time points (Alt. expos.) on the membranes, which are shown in Figure 3A. 

### 4.9. Cell Proliferation Analysis

For cell proliferation analysis, cells (2 × 10^4^ cells/well) were cultured in 12-well dishes. Cells were cultured for four days; every 24 h, the cells were trypsinized and enumerated three times using a hemocytometer, as described previously [46,96].

### 4.10. Sphere Formation Assay

For the analysis of sphere formation, Cells (4 × 10^4^ cells/well) were cultured in 35 mm non-coated dishes with SM (sphere-formation medium: serum-free DMEM/F12 supplemented with 10 μg/mL insulin (Invitrogen, Carlsbad, CA, USA), 0.4% bovine serum albumin (BSA; Sigma-Aldrich), B27-supplement (1:50; Invitrogen), and 20 ng/mL epidermal growth factor (EGF; Sigma-Aldrich)). After five days of culture, Spheres from MDA-MB-231 and KU-CSLCs were collected and stained with crystal violet (Sigma-Aldrich). The stained spheres were photographed using a Nikon Eclipse TE2000-U microscope (Nikon Instruments Inc., Melville, NY, USA) prior to colony enumerating, as described previously [46]. 

### 4.11. Wound-Healing/Migration Assay 

For wound-healing/migration analysis, 1 × 10^6^ cells were seeded in 60-mm cell culture dishes and were grown to ~90% confluence. Prior to the analysis, the cells were treated with mitomycin C (10 µg/mL) for 3–4 h at 5% CO_2_ and 37 °C, to prevent cell growth. After that, the cell monolayer was scratched with a 200 µL pipette tip to create a wound. Subsequently, the cells were washed three times with 1× PBS and images of the wound were taken using a Nikon Eclipse TE2000-U microscope (Nikon Instruments Inc.) at the indicated time-points (0, 12, and 24 h), as described previously [46,97].

### 4.12. Drug-Resistance Assay

For the analysis of drug-resistance, cells (1 × 10^5^ cells/well) were cultured in 12-well cell culture dishes and were cultured overnight at 5% CO_2_ and 37 °C. Afterward, cells were incubated with various doses of doxorubicin (DOX; 0.01, 0.1, 0.25, 0.5, and 1 µM, respectively) and were cultured for another 48 h. After 48 h incubation, cells were counted or medium was exchanged with a fresh medium containing EZ-Cytox (Daeil Lab Service, Seoul, Korea) and was incubated for an additional 3–4 h at 37 °C in an atmosphere of 5% CO_2_. The absorbance was measured at 450 nm by using a microplate reader Bio-Rad x-Mark™ spectrophotometer (Bio-Rad). The results were represented as a percentage (%) of surviving cells, as described previously [46,98].

### 4.13. Statistical Analyses

For statistical analysis, all of the experiments were independently undertaken at least three times. Data were presented as the mean ± standard deviation (SD) using Microsoft Excel 2010 software and statistical analyses were performed by ANOVA with a Bonferroni adjustment for comparing treated vs. control group by GraphPad InStat V. 3.0 software (GraphPad, San Diego, CA, USA). Results were considered to be statistically significant for *p*-values < 0.05. 

## 5. Conclusions

In conclusion, our current study has revealed that there is an inverse relationship between the expression of *KRT19* and breast cancer progression. We showed that *KRT19* was downregulated in highly aggressive patient-derived KU-CSLCs, and that the overexpression of *KRT19* could inhibit aggressiveness, including cell proliferation, sphere formation, migration, and drug resistance (overviewed in Figure 7), by suppressing the expression of the genes associated with these phenotypes. Moreover, after the knockdown of *KRT19* in relatively high *KRT19* expressing cells, MDA-MB231 induces cell proliferation, sphere formation, migration, and drug resistance and their responsive gene expression. In addition, we demonstrated that the overexpression or knockdown of *KRT19* regulated cancer stem cell reprogramming by modulating the expression of cancer stem cell markers (*ALDH1, CXCR4,* and *CD133*) as well as the level of p-Src and p-GSK3β (Tyr216). Thus, our data suggest that *KRT19* might function as a promising tumor suppressor gene for targeting highly aggressive patient-derived *ALDH1*^high^*, CXCR4*^high^*,* and *CD133*^high^ breast cancer stem cell-like cells, and could enable cancer cell reprogramming to less aggressive and more drug-sensitive cells.

## Figures and Tables

**Table 1:** Primer sequences for specific genes used for RT-PCR analysis.

Accession No.	Gene	Forward Primer (5′→3′)	Reverse Primer (5′→3′)
NM_000689.4	*ALDH1*	CTGCTGGCGACAATGGAGT	GTCAGCCCAACCTGCACAG
NM_001008540.2	*CXCR4*	CGCCACCAACAGTCAGAG	AACACAACCACCCACAAGTC
NM_001145852.1	*CD133*	GTCACCATTGACTTCTTGGTGCTGT	TGTCAGATGGAGTTACGCAGGTTTC
NM_002046.5	*GAPDH*	AATCCCATCACCATCTTCCAG	CACGATACCAAAGTTGTCATGG
NM_002276.4	*KRT19*	GCGAGCTAGAGGTGAAGATC	CGGAAGTCATCTGCAGCCA
NM_004827.2	*ABCG2*	TTATCCGTGGTGTGTCTGGAG	TCCTGCTTGGAAGGCTCTATG
NM_004996.3	*ABCC1*	GCCGGTGAAGGTTGTGTACT	CTGACGAAGCAGATGTGGAA
NM_001348946.1	*ABCB1*	GAGGAAGACATGACCAGGTA	CTGTCGCATTATAGCATGAA
NM_001285986.1	*OCT4*	GTCCCAGGACATCAAAGCTC	CTCCAGGTTGCCTCTCACTC
NM_002701.5	*OCT4A*	CGTGAAGCTGGAGAAGGAGAAGCTG	CAAGGGCCGCAGCTTACACATGTTC
NM_001285987.1	*OCT4B*	ATGCATGAGTCAGTGAACAG	CCACATCGGCCTGTGTATAT
NM_001314052.1	*KLF4*	GAACTGACCAGGCACTACCG	TTCTGGCAGTGTGGGTCATA
NM_003106.3	*SOX2*	ACACCAATCCCATCCACACT	GCAAGAAGCCTCTCCTTGAA
NM_024865.3	*NANOG*	ATACCTCAGCCTCCAGCAGA	GCAGGACTGCAGAGATTCCT
NM_002467.4	*C* *-MYC*	CTCGGATTCTCTGCTCTC	TCGCCTCTTGACATTCTC
NM_001126118.1	*P53*	GCCCAACAACACCAGCTCCT	CCTGGGCATCCTTGAGTTCC
XM_017008921.2	*N-cadherin*	TGGATGGACCTTATGTTGCT	AACACCTGTCTTGGGATCAA
NM_001205255.1	*OCLN*	CTTCAGGCAGCCTCGTTACA	TCCTCCTCCAGCTCATCACA
NM_001317185.1	*E-cadherin*	CAG CAC GTA CAC AGC CCT AA	ACC CAC CTC TAA GGC CAT CT
NM_001323654.1	*ZEB1*	GCCAATAAGCAAACGATTCTG	TTTGGCTGGATCACTTTCAAG
NM_000474.3	*TWIST1*	CTCAGCTACGCCTTCTCG	ACTGTCCATTTTCTCCTTCTCTG

## Supplementary Materials

Supplementary materials can be found at http://www.mdpi.com/1422-0067/19/5/1423/s1.

## Abbreviations

*KRT19*	cytokeratin 19
iPSC	induced pluripotent stem cell
PS	pluripotent stem
CSC	cancer stem cell
KU-CSLC	konkuk University-cancer stem cell-like cell
KRT19-oe	keratin 19-overexpression
RT-PCR	reverse transcriptase polymerase chain reaction
TF	Transcription factor
qRT-PCR	quantitative real-time-PCR
shRNA	short hairpin RNA
shKRT19	short hairpin keratin19
SCID	severe combined immunodeficiency
WB	western blot
EMT	epithelial to mesenchymal transition
MET	mesenchymal to epithelial transition
DOX	doxorubicin
STR	short tandem repeat
CDS	coding sequence
SDS-PAGE	sodium dodecyl sulfate-polyacrylamide gel electrophoresis
TBS	tris-buffered saline
PBS	phosphate-buffered saline
TBST	tris-buffered saline Tween 20
HRP	horseradish peroxidase
ECL	enhanced chemiluminescence

## References

[1] Almendro V., Marusyk A., Polyak K. (2013). Cellular Heterogeneity and Molecular Evolution in Cancer. Annu. Rev. Pathol. Mech..

[2] Ferlay J., Soerjomataram I., Dikshit R., Eser S., Mathers C., Rebelo M., Parkin D.M., Forman D., Bray F. (2015). Cancer incidence and mortality worldwide: Sources, methods and major patterns in GLOBOCAN 2012. Int. J. Cancer.

[3] You J.S., Jones P.A. (2012). Cancer genetics and epigenetics: Two sides of the same coin?. Cancer Cell.

[4] Reya T., Morrison S.J., Clarke M.F., Weissman I.L. (2001). Stem cells, cancer, and cancer stem cells. Nature.

[5] Chestkov I.V., Khomyakova E.A., Vasilieva E.A., Lagarkova M.A., Kiselev S.L. (2014). Molecular barriers to processes of genetic reprogramming and cell transformation. Biochemistry.

[6] Bogomazova A.N., Vassina E.M., Kiselev S.I., Lagarkova M.A., Lebedeva O.S., Nekrasov E.D., Panova A.V., Philonenko E.S., Khomyakova E.A., Tskhovrebova L.V. (2015). Genetic Cell Reprogramming: A New Technology for Basic Research and Applied Usage. Genetika.

[7] Hochedlinger K., Yamada Y., Beard C., Jaenisch R. (2005). Ectopic expression of Oct-4 blocks progenitor-cell differentiation and causes dysplasia in epithelial tissues. Cell.

[8] Chiou S.H., Wang M.L., Chou Y.T., Chen C.J., Hong C.F., Hsieh W.J., Chang H.T., Chen Y.S., Lin T.W., Hsu H.S. (2010). Coexpression of Oct4 and Nanog Enhances Malignancy in Lung Adenocarcinoma by Inducing Cancer Stem Cell-Like Properties and Epithelial-Mesenchymal Transdifferentiation. Cancer Res..

[9] Takahashi K., Yamanaka S. (2006). Induction of pluripotent stem cells from mouse embryonic and adult fibroblast cultures by defined factors. Cell.

[10] Monk M., Holding C. (2001). Human embryonic genes re-expressed in cancer cells. Oncogene.

[11] Zhao W., Ji X., Zhang F., Li L., Ma L. (2012). Embryonic Stem Cell Markers. Molecules.

[12] Schoenhals M., Kassambara A., De Vos J., Hose D., Moreaux J., Klein B. (2009). Embryonic stem cell markers expression in cancers. Biochem. Biophs. Res. Commun..

[13] Gillis A.J.M., Stoop H., Biermann K., van Gurp R.J., Swartzman E., Cribbes S., Ferlinz A., Shannon M., Oosterhuis J.W., Looijenga L.H.J. (2011). Expression and interdependencies of pluripotency factors LIN28, OCT3/4, NANOG and SOX2 in human testicular germ cells and tumours of the testis. Int. J. Androl..

[14] Forghanifard M.M., Khales S.A., Javdani-Mallak A., Rad A., Farshchian M., Abbaszadegan M.R. (2014). Stemness state regulators SALL4 and SOX2 are involved in progression and invasiveness of esophageal squamous cell carcinoma. Med. Oncol..

[15] Sodja E., Rijavec M., Koren A., Sadikov A., Korosec P., Cufer T. (2016). The prognostic value of whole blood SOX2, NANOG and OCT4 mRNA expression in advanced small-cell lung cancer. Radiol. Oncol..

[16] Tai S.K., Yang M.H., Chang S.Y., Chang Y.C., Li W.Y., Tsai T.L., Wang Y.F., Chu P.Y., Hsieh S.L. (2011). Persistent Kruppel-like factor 4 expression predicts progression and poor prognosis of head and neck squamous cell carcinoma. Cancer Sci..

[17] Lin C.Y., Loven J., Rahl P.B., Paranal R.M., Burge C.B., Bradner J.E., Lee T.I., Young R.A. (2012). Transcriptional Amplification in Tumor Cells with Elevated c-Myc. Cell.

[18] Xu K., Zhu Z., Zeng F. (2007). Expression and significance of Oct4 in bladder cancer. J. Huazhong Univ. Sci. Technol. Med. Sci..

[19] Hatefi N., Nouraee N., Parvin M., Ziaee S.A.M., Mowla S.J. (2012). Evaluating the Expression of Oct4 as a Prognostic Tumor Marker in Bladder Cancer. Iran. J. Basic Med. Sci..

[20] De Resende M.F., Chinen L.T.D., Vieira S., Jampietro J., da Fonseca F.P., Vassallo J., Campos L.C., Guimares G.C., Soares F.A., Rocha R.M. (2013). Prognostication of OCT4 isoform expression in prostate cancer. Tumor Biol..

[21] Rodini C.O., Suzuki D.E., Saba-Silva N., Cappellano A., de Souza J.E.S., Cavalheiro S., Toledo S.R.C., Okamoto O.K. (2012). Expression analysis of stem cell-related genes reveal OCT4 as a predictor of poor clinical outcome in medulloblastoma. J. Neuro-Oncol..

[22] Li C.G., Yan Y., Ji W.D., Bao L.L., Qian H.H., Chen L., Wu M.C., Chen H.Z., Li Z.G., Su C.Q. (2012). OCT4 Positively Regulates Survivin Expression to Promote Cancer Cell Proliferation and Leads to Poor Prognosis in Esophageal Squamous Cell Carcinoma. PLoS ONE.

[23] Ohnishi K., Semi K., Yamamoto T., Shimizu M., Tanaka A., Mitsunaga K., Okita K., Osafune K., Arioka Y., Maeda T. (2014). Premature Termination of Reprogramming In Vivo Leads to Cancer Development through Altered Epigenetic Regulation. Cell.

[24] Al-Hajj M., Wicha M.S., Benito-Hernandez A., Morrison S.J., Clarke M.F. (2003). Prospective identification of tumorigenic breast cancer cells. Proc. Natl. Acad. Sci. USA.

[25] Singh S.K., Clarke I.D., Terasaki M., Bonn V.E., Hawkins C., Squire J., Dirks P.B. (2003). Identification of a cancer stem cell in human brain tumors. Cancer Res..

[26] Ricci-Vitiani L., Lombardi D.G., Pilozzi E., Biffoni M., Todaro M., Peschle C., De Maria R. (2007). Identification and expansion of human colon-cancer-initiating cells. Nature.

[27] Bonnet D., Dick J.E. (1997). Human acute myeloid leukemia is organized as a hierarchy that originates from a primitive hematopoietic cell. Nat. Med..

[28] Collins A.T., Berry P.A., Hyde C., Stower M.J., Maitland N.J. (2005). Prospective identification of tumorigenic prostate cancer stem cells. Cancer Res..

[29] Clarke M.F., Dick J.E., Dirks P.B., Eaves C.J., Jamieson C.H., Jones D.L., Visvader J., Weissman I.L., Wahl G.M. (2006). Cancer stem cells––Perspectives on current status and future directions: AACR Workshop on cancer stem cells. Cancer Res..

[30] Pardal R., Clarke M.F., Morrison S.J. (2003). Applying the principles of stem-cell biology to cancer. Nat. Rev. Cancer.

[31] Ignatiadis M., Xenidis N., Perraki M., Apostolaki S., Politaki E., Kafousi M., Stathopoulos E.N., Stathopoulou A., Lianidou E., Chlouverakis G. (2007). Different prognostic value of cytokeratin-19 mRNA positive circulating tumor cells according to estrogen receptor and HER2 status in early-stage breast cancer. J. Clin. Oncol..

[32] Bozionellou V., Mavroudis D., Perraki M., Papadopoulos S., Apostolaki S., Stathopoulos E., Stathopoulou A., Lianidou E., Georgoulias V. (2004). Trastuzumab administration can effectively target chemotherapy-resistant cytokeratin-19 messenger RNA–positive tumor cells in the peripheral blood and bone marrow of patients with breast cancer. Clin. Cancer Res..

[33] Fradette J., Germain L., Seshaiah P., Coulombe P.A. (1998). The type I keratin 19 possesses distinct and context-dependent assembly properties. J. Biol. Chem..

[34] Coulombe P.A., Wong P. (2004). Cytoplasmic intermediate filaments revealed as dynamic and multipurpose scaffolds. Nat. Cell Biol..

[35] Hendrix M.J.C., Seftor E.A., Chu Y.W., Trevor K.T., Seftor R.E.B. (1996). Role of intermediate filaments in migration, invasion and metastasis. Cancer Metast. Rev..

[36] Kawai T., Yasuchika K., Ishii T., Katayama H., Yoshitoshi E.Y., Ogiso S., Kita S., Yasuda K., Fukumitsu K., Mizumoto M. (2015). Keratin 19, a Cancer Stem Cell Marker in Human Hepatocellular Carcinoma. Clin. Cancer Res..

[37] Govaere O., Petz M., Wouters J., Vandewynckel Y.-P., Scott E.J., Topal B., Nevens F., Verslype C., Anstee Q.M., Van Vlierberghe H. (2017). The PDGFRα-laminin B1-keratin 19 cascade drives tumor progression at the invasive front of human hepatocellular carcinoma. Oncogene.

[38] Govaere O., Komuta M., Berkers J., Spee B., Janssen C., de Luca F., Katoonizadeh A., Wouters J., van Kempen L.C., Durnez A. (2014). Keratin 19: A key role player in the invasion of human hepatocellular carcinomas. Gut.

[39] Kim H., Choi G.H., Na D.C., Ahn E.Y., Kim G.I., Lee J.E., Cho J.Y., Yoo J.E., Choi J.S., Park Y.N. (2011). Human hepatocellular carcinomas with “Stemness”-related marker expression: Keratin 19 expression and a poor prognosis. Hepatology.

[40] Ju J.H., Oh S., Lee K.M., Yang W., Nam K.S., Moon H.G., Noh D.Y., Kim C.G., Park G., Park J.B. (2015). Cytokeratin19 induced by HER2/ERK binds and stabilizes HER2 on cell membranes. Cell Death Differ..

[41] Ohtsuka T., Sakaguchi M., Yamamoto H., Tomida S., Takata K., Shien K., Hashida S., Miyata-Takata T., Watanabe M., Suzawa K. (2016). Interaction of cytokeratin 19 head domain and HER2 in the cytoplasm leads to activation of HER2-Erk pathway. Sci. Rep..

[42] Lapouge G., Youssef K.K., Vokaer B., Achouri Y., Michaux C., Sotiropoulou P.A., Blanpain C. (2011). Identifying the cellular origin of squamous skin tumors. Proc. Natl. Acad. Sci. USA.

[43] Means A.L., Xu Y.W., Zhao A.Z., Ray K.C., Gu G.Q. (2008). A CK19(CreERT) knockin mouse line allows for conditional DNA recombination in epithelial cells in multiple endodermal organs. Genesis.

[44] Asfaha S., Hayakawa Y., Muley A., Stokes S., Graham T.A., Ericksen R.E., Westphalen C.B., von Burstin J., Mastracci T.L., Worthley D.L. (2015). Krt19(+)/Lgr5(-) Cells Are Radioresistant Cancer-Initiating Stem Cells in the Colon and Intestine. Cell Stem Cell.

[45] Kabir N.N., Ronnstrand L., Kazi J.U. (2014). Keratin 19 expression correlates with poor prognosis in breast cancer. Mol. Biol. Rep..

[46] Saha S., Choi H., Kim B., Dayem A., Yang G., Kim K., Yin Y., Cho S. (2017). KRT19 directly interacts with β-catenin/RAC1 complex to regulate NUMB-dependent NOTCH signaling pathway and breast cancer properties. Oncogene.

[47] Ju J.H., Yang W., Lee K.M., Oh S., Nam K., Shim S., Shin S.Y., Gye M.C., Chu I.S., Shin I. (2013). Regulation of Cell Proliferation and Migration by Keratin19-Induced Nuclear Import of Early Growth Response-1 in Breast Cancer Cells. Clin. Cancer Res..

[48] Bambang I.F., Lu D., Li H.P., Chiu L.L., Lau Q.C., Koay E., Zhang D.H. (2009). Cytokeratin 19 regulates endoplasmic reticulum stress and inhibits ERp29 expression via p38 MAPK/XBP-1 signaling in breast cancer cells. Exp. Cell Res..

[49] Perou C.M., Sorlie T., Eisen M.B., van de Rijn M., Jeffrey S.S., Rees C.A., Pollack J.R., Ross D.T., Johnsen H., Akslen L.A. (2000). Molecular portraits of human breast tumours. Nature.

[50] Miki J., Furusato B., Li H., Gu Y., Takahashi H., Egawa S., Sesterhenn I.A., McLeod D.G., Srivastava S., Rhim J.S. (2007). Identification of putative stem cell markers, CD133 and CXCR4, in hTERT-immortalized primary nonmalignant and malignant tumor-derived human prostate epithelial cell lines and in prostate cancer specimens. Cancer Res..

[51] Douville J., Beaulieu R., Balicki D. (2009). ALDH1 as a Functional Marker of Cancer Stem and Progenitor Cells. Stem Cells Dev..

[52] Martini M., De Santis M.C., Braccini L., Gulluni F., Hirsch E. (2014). PI3K/AKT signaling pathway and cancer: An updated review. Ann. Med..

[53] Zhang X., Jiang G., Sun M., Zhou H., Miao Y., Liang M., Wang E., Zhang Y. (2017). Cytosolic THUMPD1 promotes breast cancer cells invasion and metastasis via the AKT-GSK3-Snail pathway. Oncotarget.

[54] Sun Y., Liu W.Z., Liu T., Feng X., Yang N., Zhou H.F. (2015). Signaling pathway of MAPK/ERK in cell proliferation, differentiation, migration, senescence and apoptosis. J. Recept. Signal. Transduct. Res..

[55] Kim R.K., Cui Y.H., Yoo K.C., Kim I.G., Lee M., Choi Y.H., Suh Y., Lee S.J. (2015). Radiation promotes malignant phenotypes through SRC in breast cancer cells. Cancer Sci..

[56] Malki A., Elbayaa R.Y., Ashour H.M.A., Loffredo C.A., Youssef A.M. (2015). Novel thiosemicarbazides induced apoptosis in human MCF-7 breast cancer cells via JNK signaling. J. Enzym. Inhib. Med. Chem..

[57] Yu F., Li J., Chen H., Fu J., Ray S., Huang S., Zheng H., Ai W. (2011). Kruppel-like factor 4 (KLF4) is required for maintenance of breast cancer stem cells and for cell migration and invasion. Oncogene.

[58] Leis O., Eguiara A., Lopez-Arribillaga E., Alberdi M.J., Hernandez-Garcia S., Elorriaga K., Pandiella A., Rezola R., Martin A.G. (2012). Sox2 expression in breast tumours and activation in breast cancer stem cells. Oncogene.

[59] Rhodes D.R., Yu J.J., Shanker K., Deshpande N., Varambally R., Ghosh D., Barrette T., Pandey A., Chinnaiyan A.M. (2004). ONCOMINE: A cancer microarray database and integrated data-mining platform. Neoplasia.

[60] Rhodes D.R., Kalyana-Sundaram S., Mahavisno V., Varambally R., Yu J.J., Briggs B.B., Barrette T.R., Anstet M.J., Kincead-Beal C., Kulkarni P. (2007). Oncomine 3.0: Genes, pathways, and networks in a collection of 18,000 cancer gene expression profiles. Neoplasia.

[61] Mansour S.F., Atwa M.M. (2015). Clinicopathological Significance of CD133 and ALDH1 Cancer Stem Cell Marker Expression in Invasive Ductal Breast Carcinoma. Asian Pac. J. Cancer Prev..

[62] Roudi R., Korourian A., Shariftabrizi A., Madjd Z. (2015). Differential Expression of Cancer Stem Cell Markers ALDH1 and CD133 in Various Lung Cancer Subtypes. Cancer Invest..

[63] Cioffi M., D’Alterio C., Camerlingo R., Tirino V., Consales C., Riccio A., Ierano C., Cecere S.C., Losito N.S., Greggi S. (2015). Identification of a distinct population of CD133+CXCR4+ cancer stem cells in ovarian cancer. Sci. Rep..

[64] Tu Z.B., Xie S.P., Xiong M., Liu Y.C., Yang X.Y., Tembo K.M., Huang J., Hu W.D., Huang X.X., Pan S. (2017). CXCR4 is involved in CD133-induced EMT in non-small cell lung cancer. Int. J. Oncol..

[65] Sun Y., Yoshida T., Okabe M., Zhou K.X., Wang F., Soko C., Saito S., Nikaido T. (2017). Isolation of Stem-Like Cancer Cells in Primary Endometrial Cancer Using Cell Surface Markers CD133 and CXCR4. Transl. Oncol..

[66] Adorno-Cruz V., Kibria G., Liu X., Doherty M., Junk D.J., Guan D., Hubert C., Venere M., Mulkearns-Hubert E., Sinyuk M. (2015). Cancer stem cells: Targeting the roots of cancer, seeds of metastasis, and sources of therapy resistance. Cancer Res..

[67] Li C.G., Zhu M.L., Lou X.L., Liu C.Y., Chen H.Z., Lin X.J., Ji W.D., Li Z.G., Su C.Q. (2017). Transcriptional factor OCT4 promotes esophageal cancer metastasis by inducing epithelial-mesenchymal transition through VEGF-C/VEGFR-3 signaling pathway. Oncotarget.

[68] Liu K.C., Xie F., Gao A.D., Zhang R., Zhang L., Xiao Z.W., Hu Q., Huang W.F., Huang Q.J., Lin B.S. (2017). SOX2 regulates multiple malignant processes of breast cancer development through the SOX2/miR-181a-5p, miR-30e-5p/TUSC3 axis. Mol. Cancer.

[69] Murgai M., Ju W., Eason M., Kline J., Kaplan R.N. (2017). KLF4-dependent perivascular plasticity enhances pre-metastatic niche formation and metastasis. Nat. Med..

[70] Guo T., Kong J., Liu Y., Li Z., Xia J., Zhang Y., Zhao S., Li F., Li J., Gu C. (2017). Transcriptional activation of NANOG by YBX1 promotes lung cancer stem-like properties and metastasis. Biochem. Biophys. Res. Commun..

[71] Chen Y.-C., Hsu H.-S., Chen Y.-W., Tsai T.-H., How C.-K., Wang C.-Y., Hung S.-C., Chang Y.-L., Tsai M.-L., Lee Y.-Y. (2008). Oct-4 expression maintained cancer stem-like properties in lung cancer-derived CD133-positive cells. PLoS ONE.

[72] Saigusa S., Tanaka K., Toiyama Y., Yokoe T., Okugawa Y., Ioue Y., Miki C., Kusunoki M. (2009). Correlation of CD133, OCT4, and SOX2 in rectal cancer and their association with distant recurrence after chemoradiotherapy. Ann. Surg. Oncol..

[73] Mu P., Zhang Z., Benelli M., Karthaus W.R., Hoover E., Chen C.-C., Wongvipat J., Ku S.-Y., Gao D., Cao Z. (2017). SOX2 promotes lineage plasticity and antiandrogen resistance in TP53-and RB1-deficient prostate cancer. Science.

[74] Riz I., Hawley T.S., Hawley R.G. (2015). KLF4-SQSTM1/p62-associated prosurvival autophagy contributes to carfilzomib resistance in multiple myeloma models. Oncotarget.

[75] Tsai L.L., Yu C.C., Chang Y.C., Yu C.H., Chou M.Y. (2011). Markedly increased Oct4 and Nanog expression correlates with cisplatin resistance in oral squamous cell carcinoma. J. Oral Pathol. Med..

[76] Moreno-Bueno G., Portillo F., Cano A. (2008). Transcriptional regulation of cell polarity in EMT and cancer. Oncogene.

[77] De Figueiredo-Pontes L.L., Pintao M.C.T., Oliveira L.C.O., Dalmazzo L.F.F., Jacomo R.H., Garcia A.B., Falcao R.P., Rego E.M. (2008). Determination of P-glycoprotein, MDR-related protein 1, breast cancer resistance protein, and lung-resistance protein expression in leukemic stem cells of acute myeloid leukemia. Cytom. B-Clin. Cytom..

[78] Goding C.R., Pei D., Lu X. (2014). Cancer: Pathological nuclear reprogramming?. Nat. Rev. Cancer.

[79] (2013). Nuclear reprogramming and the cancer genome. Nat. Genet..

[80] Kurebayashi J., Kanomata N., Moriya T., Kozuka Y., Watanabe M., Sonoo H. (2010). Preferential antitumor effect of the Src inhibitor dasatinib associated with a decreased proportion of aldehyde dehydrogenase 1-positive cells in breast cancer cells of the basal B subtype. BMC Cancer.

[81] Bolos V., Gasent J.M., Lopez-Tarruella S., Grande E. (2010). The dual kinase complex FAK-Src as a promising therapeutic target in cancer. OncoTargets Ther..

[82] Orgaz J.L., Pandya P., Dalmeida R., Karagiannis P., Sanchez-Laorden B., Viros A., Albrengues J., Nestle F.O., Ridley A.J., Gaggioli C. (2014). Diverse matrix metalloproteinase functions regulate cancer amoeboid migration. Nat. Commun..

[83] Thakur R., Trivedi R., Rastogi N., Singh M., Mishra D.P. (2015). Inhibition of STAT3, FAK and Src mediated signaling reduces cancer stem cell load, tumorigenic potential and metastasis in breast cancer. Sci. Rep..

[84] Paladino D., Yue P., Furuya H., Acoba J., Rosser C.J., Turkson J. (2016). A novel nuclear Src and p300 signaling axis controls migratory and invasive behavior in pancreatic cancer. Oncotarget.

[85] Ren H., Fang J., Ding X.J., Chen Q.Y. (2016). Role and inhibition of Src signaling in the progression of liver cancer. Open Life Sci..

[86] Goc A., Al-Husein B., Katsanevas K., Steinbach A., Lou U., Sabbineni H., DeRemer D.L., Somanath P.R. (2014). Targeting Src-mediated Tyr216 phosphorylation and activation of GSK-3 in prostate cancer cells inhibit prostate cancer progression in vitro and in vivo. Oncotarget.

[87] Jope R.S., Yuskaitis C.J., Beurel E. (2007). Glycogen synthase kinase-3 (GSK3): Inflammation, diseases, and therapeutics. Neurochem. Res..

[88] Hughes K., Nikolakaki E., Plyte S.E., Totty N.F., Woodgett J.R. (1993). Modulation of the glycogen synthase kinase-3 family by tyrosine phosphorylation. EMBO J..

[89] Kaidanovich-Beilin O., Woodgett J.R. (2011). GSK-3: Functional Insights from Cell Biology and Animal Models. Front. Mol. Neurosci..

[90] Lánczky A., Nagy Á., Bottai G., Munkácsy G., Szabó A., Santarpia L., Győrffy B. (2016). miRpower: A web-tool to validate survival-associated miRNAs utilizing expression data from 2178 breast cancer patients. Breast Cancer Res. Treat..

[91] Dayem A.A., Kim B., Gurunathan S., Choi H.Y., Yang G., Saha S.K., Han D., Han J., Kim K., Kim J.H. (2014). Biologically synthesized silver nanoparticles induce neuronal differentiation of SH-SY5Y cells via modulation of reactive oxygen species, phosphatases, and kinase signaling pathways. Biotechnol. J..

[92] Nishikawa S., Konno M., Hamabe A., Hasegawa S., Kano Y., Fukusumi T., Satoh T., Takiguchi S., Mori M., Doki Y. (2015). Surgically resected human tumors reveal the biological significance of the gastric cancer stem cell markers CD44 and CD26. Oncol. Lett..

[93] Feldman J.P., Goldwasser R., Mark S., Schwartz J., Orion I. (2009). A mathematical model for tumor volume evaluation using two-dimensions. J. Appl. Quant. Methods.

[94] Kutner R.H., Zhang X.Y., Reiser J. (2009). Production, concentration and titration of pseudotyped HIV-1-based lentiviral vectors. Nat. Protoc..

[95] Saha S.K., Yin Y., Kim K., Yang G.M., Dayem A.A., Choi H.Y., Cho S.G. (2017). Valproic Acid Induces Endocytosis-Mediated Doxorubicin Internalization and Shows Synergistic Cytotoxic Effects in Hepatocellular Carcinoma Cells. Int. J. Mol. Sci..

[96] Moon S.H., Kim D.K., Cha Y., Jeon I., Song J., Park K.S. (2013). PI3K/Akt and Stat3 signaling regulated by PTEN control of the cancer stem cell population, proliferation and senescence in a glioblastoma cell line. Int. J. Oncol..

[97] Kujawski M., Kortylewski M., Lee H., Herrmann A., Kay H., Yu H. (2008). Stat3 mediates myeloid cell-dependent tumor angiogenesis in mice. J. Clin. Investig..

[98] Hua G.J., Liu Y.P., Li X.Y., Xu P.R., Luo Y.C. (2014). Targeting glucose metabolism in chondrosarcoma cells enhances the sensitivity to doxorubicin through the inhibition of lactate dehydrogenase-A. Oncol. Rep..

