# Supplementary material for: Cytokeratin 19 (KRT19) has a Role in the Reprogramming of Cancer Stem Cell-Like Cells to Less Aggressive and More Drug-Sensitive Cells
Source: Int J Mol Sci. 2018 May 9;19(5):1423. doi: 10.3390/ijms19051423 (PMC5983664; doi:10.3390/ijms19051423)
Supplement: Supplementary file 1 [file ijms-19-01423-s001.pdf]

Supplementary Information

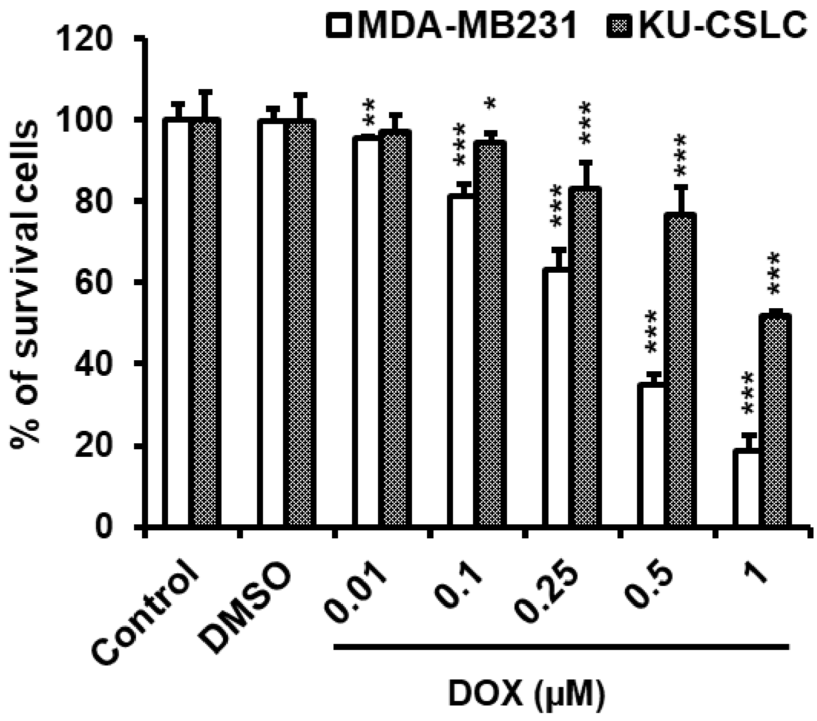

Figure S1. The dose dependent effects of doxorubicin (DOX) on cell viability of breast cancer cells. The viability of MDA-MB231 and KU-CSLC cells was determined by EZ-Cytox assay after 48-h exposure to the indicated concentration of DOX. *$p < 0.05$; **$p < 0.01$; ***$p < 0.001$.
